# What kind of science for dual diagnosis? A pragmatic examination of the enactive approach to psychiatry

**DOI:** 10.3389/fpsyg.2022.825701

**Published:** 2022-07-18

**Authors:** Jonathan Led Larsen, Katrine Schepelern Johansen, Mimi Yung Mehlsen

**Affiliations:** ^1^Sankt Hans Hospital, Roskilde, Denmark; ^2^Department of Psychology and Behavioural Sciences, School of Business and Social Sciences, Aarhus University, Aarhus, Denmark

**Keywords:** dual diagnosis, enactivism, cross-disciplinarity, cannabis use, pragmatism, psychosis, qualitative, longitudinal

## Abstract

The recommended treatment for *dual diagnosis* - the co-occurrence of substance use and another mental disorder - requires seamless integration of the involved disciplines and services. However, no integrative framework exists for communicating about dual diagnosis cases across disciplinary or sectoral boundaries. We examine if *Enactive Psychiatry* may bridge this theoretical gap. We evaluate the enactive approach through a two-step pragmatic lens: Firstly, by taking a historical perspective to describe more accurately how the theoretical gap within the field of dual diagnosis initially developed. Secondly, by applying the Enactive Psychiatry approach to data from a longitudinal study on the trajectory of cannabis use in psychosis disorders. By applying the theory rather than simply presenting it, we position ourselves better to evaluate whether it may assist the purpose of achieving a more expedient pragmatic “grip” on the field of dual diagnosis. In our discussion, we suggest that this may very well be the case. Finally, we consider the enactive approach as one of a small handful of new theories of mental disorders that draw on systems thinking and ecological psychology, and discuss whether they have the potential for a wider progressive problemshift within psychiatry. The case in favor of such potential, we argue, is less strong unless the role of complexity, similar to that seen within the dual diagnosis field, may be demonstrated for other fields of clinical practice.

## Introduction

Roughly speaking, three different lenses have been used to understand psychiatric diagnoses. Either diagnoses refer to natural entities existing independently of any classification system (realism), they are useful categories (pragmatism), or they depend crucially on the socio-cultural processes that goes into defining what is normal and abnormal (social constructionism). In recent decades, psychiatry has tended to retreat from realism into pragmatism (Kendler, 2016). However, pragmatism, when used as a lens to perceive diagnoses, is faced by a dilemma when attempting to explain what makes a diagnostic system practical: Either practicality is rooted in a match with reality, which indicates realism, or it must be shaped by other forces, which pushes us toward constructionism (Hartner and Theurer, 2018). However, pragmatism can also be understood not as a lens allowing us to interpret already established diagnoses but as a scientific approach to bottom-up problem solving. Here, we utilize a pragmatic and theoretically informed approach to empirically examine a particular corner of psychiatry - that of the dual diagnosis field.

Dual diagnosis - also referred to as co-occurring addictive and psychiatric disorders - is an outlier in the psychiatric treatment systems of most Western countries (Dom and Moggi, 2015). Patients falling into the dual diagnosis category are challenging for existing treatment systems and research frameworks because they often experience many interacting problems, such as family conflicts, challenges in the educational system, a poor economy, a history of trauma, somatic health problems, lack of employment and so forth (Drake and Green, 2013). Treating dual diagnosis is additionally complicated by structural challenges: In most western countries, mental health services and addiction treatment systems are separated (Dom and Moggi, 2015). Furthermore, in a Danish context, where this research was conducted, a paradigmatic gap exists between a medically oriented psychiatric system and a psychosocially oriented addiction treatment system (Johansen, 2018). This state of affairs is unfortunate because it biases treatment toward either sequential or parallel treatment, whereas recommended dual diagnosis treatment is integrative (NICE, 2011; Helsedirektoratet, 2012). Sequential treatment refers to one type of treatment (e.g., psychiatric treatment) being followed by another (addiction treatment). Parallel treatment involves following two separate lines of treatment simultaneously, whereas the optimal treatment mode, integrative treatment, ideally offers seamless cooperation between disciplines and between sectors, preferably organized through a single point of access. In this article, we apply a novel theoretical approach to psychiatry to the field of dual diagnosis in order to explore the theory’s potential as a cross-disciplinary framework that may be developed to support integrated treatment efforts.

Recent years have witnessed several new theoretical approaches to psychiatry that draw on systems theory, embodied cognition and ecological psychology to provide an integrative framework for psychiatry, thus providing us with a way of addressing the diverse kinds of factors involved in the lives of patients with a dual diagnosis. These theories are coined, e.g., *Neuroecosocial mental health* (Rose et al., 2021), *Ecosocial psychiatry* (Kirmayer, 2019), *Relational analysis of phenomena* (Nielsen and Ward, 2020) and *Enactive psychiatry* (Fuchs, 2018; de Haan, 2020b). However, they mainly stay on a level of abstraction that the philosopher of science Imre Lakatos called “hard core,” meaning that their assumptions cannot directly be falsified. According to Lakatos, a hard core theory may, nevertheless, be judged by its ability to generate research questions and move the field forward, i.e., whether it produces a “progressive problemshift” that leads to theoretical and/or empirical progress (Lakatos, 1980, p. 48). We single out the Enactive Psychiatry approach and move it out of the abstract “hard core” sphere by applying its perspective to empirical material from a longitudinal, qualitative study of cannabis use in psychosis. Our intent is not to “prove” the approach (as this would be futile), but to examine if it is useful in making sense of the empirical material in a way that can potentially help move the dual diagnosis field forward.

This *Hypothesis and Theory* article is executed in two parts; according to the pragmatic approach, as we use it here, what first sets in motion a sequence of problem solving is an observed difficulty [“a felt difficulty” (Dewey, 1910, p. 72)], which then triggers a closer examination of the problem at hand [“its location and definition” (Dewey, 1910, p. 72)]. The next step is contemplating how to solve the problem [“suggestion of possible solution” (Dewey, 1910, p. 72)], for which a theory is needed (Lewis, 2007, p. 302).

We refer to Dewey’s steps in problem solving but draw on particular work in Science and Technology Studies for our overall understanding of pragmatism. Here theory is understood as entwined with performance in a fundamental way: knowledge is developed through so-called performative “dances of agency” between researcher and the object of study (Pickering, 1995, 2010, 2014). Co-evolving theory and practice then develops into more mature, practical “grips” on particular challenges - in psychiatry these are generally either pharmacological, psychotherapeutic or environmental in nature. This version of pragmatism importantly helps us acknowledge different grips as having merit simultaneously while at the same time deflating search for a single universal truth or solution for a given difficulty. Thus, classic conflicts, e.g., between a biological and a psychological approach to mental disorders (Luhrmann, 2000), is rephrased as a question of different grips necessarily supported by different conceptualizations and bodies of knowledge. Theory and models that are a poor fit to the phenomena under scrutiny can constrain research and practice in unhelpful ways which, it has been suggested, may be the case with present diagnostic systems (Hyman, 2010). On the other hand, constructive synergy between theorizing and practice may be helpful. In parallel to the steps involved in problem solving this pragmatic framework alerts us to consider first how the dual diagnosis field is constituted, and then how theoretical considerations may help improve our grip.

In the first part of the article, we sketch how the field of dual diagnosis emerged. Of course, how the field evolved cannot be answered in a definitive way. Nevertheless, we provide one interpretation of the field’s history, which is supported by other researchers in the field, because it helps us define the problem we wish to address. Hence, what we intend to address is not dual diagnosis problems in an individual sense but the wider situation (Clarke et al., 2017) surrounding dual diagnosis problems, which constitute a field that is underserved and widely considered challenging (Carrà et al., 2015). We conclude part one by defining the problem within the field of dual diagnosis that we wish to address in the second part of the article.

The second part of the article introduces the Enactive Psychiatry approach to mental disorders and simultaneously uses this approach to engage with empirical material from the field of dual diagnosis. Theory, as we understand it here (Lewis, 2007, pp. 302–303), helps us organize data and also guides how we engage with a difficulty. By introducing the theory in tandem with the empirical material, we examine what kinds of phenomena the theory allows us to see in the material. This also helps us move one of the new theories of psychiatry into concrete territory. Pragmatically, the success of research and knowledge creation is measured in terms of how it helps us handle our problem of concern; and by engaging with both theory and data, we are in a better position to evaluate its usefulness.

After having presented our findings, we discuss the potential of the new theories re the state of the dual diagnosis field and the central challenge that we have defined. In closing, we turn to considering whether the kind of science proposed here for the field of dual diagnosis may also hold potential for the wider psychiatric field.

## Dual diagnosis: Emergence of a field

In the following, we sketch the emergence of the dual diagnosis field. As mentioned, this narrative is one of several possible interpretations. Nevertheless, from the point of view of the pragmatic approach employed in this article, understanding the historical and contextual circumstances works in two ways: Firstly, it explicates our understanding of why the field of dual diagnosis has remained a major challenge despite 30 years of research on the topic; and, secondly, it helps qualify the concern within the field, which we intend to address using the Enactive Psychiatry approach. The development we sketch is focused on Western countries in general but also refers to specific Danish circumstances in order to provide concrete examples.

### Creating the conditions for dual diagnosis

In some presentations, the field of dual diagnosis is simply and unproblematically defined as the simultaneous presence of two mental disorders, one of which is an addiction disorder, while the other is not (Mueser et al., 2013). Whereas the concept of dual diagnosis was introduced in the late 1980s (Minkoff, 1989; Drake and Wallach, 2000), it did not develop in a vacuum but was made relevant by a mosaic of preceding developments - none of which, in themselves, can be considered the central driver (Schmetzer, 2007). Following the American coining of the dual diagnosis term, the corresponding Danish term, “dobbeltdiagnose,” was introduced academically in Denmark in 1994 (Jessen-Petersen, 1994).

In the decades leading up to the emergence of the field of dual diagnosis, both psychiatry and society changed immensely. With the introduction of the third edition of the Diagnostic and Statistical manual (DSM-III) in 1980, psychiatry moved from a psychoanalytic and prototypically oriented diagnostic system to a purportedly atheoretical polythetic system (Aftab and Ryznar, 2021). During this process, addiction changed from being mainly considered secondary to other psychopathologies to being considered a primary disorder in its own right (Minkoff, 1989; Robinson and Adinoff, 2016). This development helped set the scene for the field of dual diagnosis by suggesting the existence of two separate disorders (Glaser, 1993) - the order and respective importance of which have been a topic of much discussion. This development also facilitated epidemiologic research which, through screening and questionnaire studies, demonstrated prevalence rates of dual diagnosis in excess of 50% in psychiatric populations (Regier et al., 1990).

At the same time, social circumstances changed considerably. Earlier in the century, the use of psychoactive drugs (alcohol aside) had been connected mostly to initial medical use (of morphine) turned to addiction in middle class individuals (Glaser, 1993). However, the 1940s and onward saw the rise of a new type of drug use among predominantly young, socially marginalized people (Houborg, 2014). Here, drug use and social relationships started to be combined into subcultures often fueled by cultural momentum and culminating rhetorically in the 1960’s call to use drugs to achieve emancipation (Wesson, 2011). In an American context, the number of people who had tried drugs changed from around 2% at the start of the 1960s to around 50% in the 1980s (Schmetzer, 2007, pp. 97–98).

Furthermore, life circumstances changed immensely in Western countries where rising wealth was combined with the emergence of welfare states with large public sectors, which became increasingly occupied with creating productive citizens who could join the workforce. When the expansive and countercultural ethos of the 1960s and 1970s subsided, new ideals about personal development came into being: Whereas the countercultural ideals of personhood were concerned with self-development understood as introspective contemplation supported by social practices, the new ideal was the self-reliant entrepreneur who equated her worth with her material possessions and her place in the hierarchy (Davies, 2011) - a notion of worth that those on the precarious margins of society cannot match (Standing, 2015).

In short, in the decades leading up to the emergence of the field of dual diagnosis, illegal substances had become widespread in Western societies, partly carried by a countercultural ethos. At the same time, a period of socially oriented cultural diversification was being replaced by an individualistic and materialistic orientation where the still evolving state welfare systems focused increasingly on preparing citizens to join the workforce, reducing the worth of the ‘unfit’. Meanwhile, a new type of diagnostic system, introduced with the DSM-III, that was supposedly more precise, laid the ground for identifying a large number of people with dual diagnosis in epidemiological studies (Glaser, 1993, p. 50). In the context of the dual diagnosis field, it is also of importance that although the new diagnostic systems were atheoretical in nature, the change, nevertheless, signaled a renewed biomedical focus (Harrington, 2019) that tended to decontextualize dual diagnosis problems while accentuating individual biology (Drake and Wallach, 2000). The combination of substances, marginalization and individualization arguably had a bigger impact than each of these factors in isolation: Substances offer the marginalized relief; and because mental disorders are largely understood as disconnected from societal circumstances, political and economic corrections are considered unnecessary, while the search for solutions circles back to individual biology and psychology (Marecek and Lafrance, 2021). Two other major structural developments also underpinned the emerging field of dual diagnosis.

### Fragmentation of services and knowledge base

In a Danish context, while the public sector was growing, so was concern about drug use, and the 1970s witnessed a call for specialized addiction services. However, no agreement on how to understand the phenomenon of social drug use had crystallized. Instead, on the one hand, a biologically anchored interpretation saw addiction as a socially transmitted disease and pointed to isolation - preferably on an island - as the right course of action (Houborg, 2008, pp. 199–200). Meanwhile, on the other hand, a sociological and psychological interpretation saw addiction as a derailed process of socialization, which could be rectified through learning and resocialisation back into society (Winsløw, 1984). Even focusing on a Danish context, the details of the process are fuzzy, but the results were not: in the 1970s, addiction treatment was politically placed in the social sector thus creating an organizational gap to the psychiatric services - a development which is generelly mirrored in other western countries (Mueser et al., 2013; Roberts and Maybery, 2014; van der Stel, 2015).

Furthermore, this was not an organizational gap exclusively but also a paradigmatic gap of methods and knowledge. Although adopting the biopsychosocial model by name, the health-based psychiatric system to this day emphasizes the biological aspect of psychiatric disorders, is more formal and struggles to involve patients as central agents. Opposite this, the addiction treatment system is overall more psychosocially oriented and loosely organized, allowing the client a different role while being less uniform with respect to the standard of its services (Roberts and Jones, 2012). This characterization of the two systems is supported by empirical findings (Russell and Evans, 2009). Also, addiction services are not mandated to use coercion, whereas the psychiatric system is and it struggles with limited success to reduce its use of forced treatment (Sundhedsstyrelsen, 2021) - a difference that adds to the gap between services, particularly, perhaps, in the eyes of its users.

The overall effect of separating psychiatric services and addiction treatment is that dual diagnosis patients do not belong in any one system, but are left to straddle services and navigate different professional orientations, including different legal areas, while no one service system is responsible for establishing continuity of treatment; nor does a shared, integrative framework exist for communicating across these gaps (Ness et al., 2014).

In relation to psychiatry’s knowledge base, the field of dual diagnosis has proven to be a poor fit. This is so because psychiatry adopts a hierarchy of evidence with the randomized controlled trial (and systematic reviews of these) placed at the top, has narrow research designs and prioritizes single disorders without confounding issues. In contrast hereto, the field of dual diagnosis is, per definition, complex and involves heterogeneous processes (Roberts, 2014). Thus, treatment recommendations largely rest on combinations of treatments recommended for simpler problem complexes.

### Deinstitutionalisation: A change in need of theory

The most fundamental change for psychiatry in the past 50 years, however, permeates psychiatric practice every day but is seldomly discussed as a factor that should influence the theories and models underpinning psychiatric practice and research. In a Danish context, from the 1970s (earlier in United States and Italy), the large psychiatric asylums were closed and the number of beds for inpatient stays was reduced from two per thousand citizens in 1970 to 0.5 in 2007 (Bengtsson, 2011). It is probably too early to understand the importance of deinstitutionalization in full as it is arguably a process that is still unfolding (Kritsotaki et al., 2016), but deinstitutionalisation is nevertheless considered as central for the emergence of the dual diagnosis field (Drake and Wallach, 2000; Schmetzer, 2007).

What the initial drivers of the deinstitutionalisation process were is a contested matter, and it is reasonable to assume a complex mix of different developments. In a historic overview of the deinstitutionalization movement several strands are suggested, none of them considered primary (Kritsotaki et al., 2016):

• A budding social psychiatry understanding mental disorders as caused by environmental factors such as poverty, racism and violence which suggested an alternative to the psychiatric institutions.
• The introduction of new psychopharmacological treatments such as chlorpromazine which helped boost confidence in effective treatments deployable outside institutions.
• The perception among decision-makers that an outpatient-centered treatment system would be less expensive.
• A rising critique of institutional psychiatry arguing that the institutions were part of the problem rather than part of the solution, e.g., by cutting patients off from the rest of society and compromising their potential for self-determination.

**FIGURE 1:**
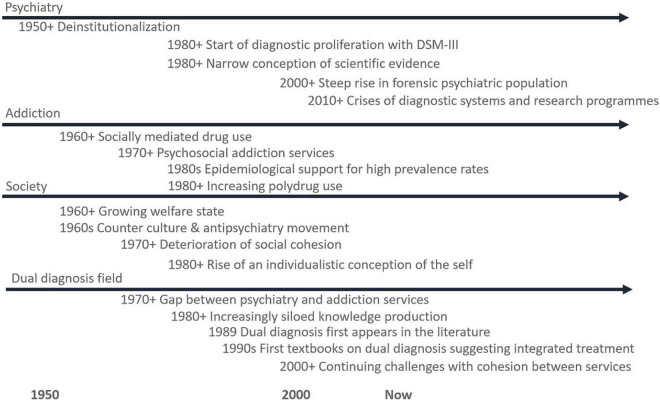
A timeline of important developments leading up to the emergence of the dual diagnosis field. Most developments are difficult to pinpoint to a specific year, so timing is indicated broadly. References are found in the main text.

However, the history of deinstitutionalisation will be laid out in the future, the transition to outpatient treatment coincided with the rise in the availability of illegal substances while a lack of adequately developed community services added to the vulnerability of patients now living in the community. Some authors see this combination as a direct precursor to the field of dual diagnosis, the rise in homelessness among people with psychiatric disorders and the rising number of forensic psychiatric patients (Nordentoft, 1990; Drake and Wallach, 2000; Roberts, 2014). In a 1990 analysis of the deinstitutionalisation process both in a Danish and international context, a prominent Danish psychiatrist, who has published extensively within the field of dual diagnosis, concluded that a pressing need exists for “outreach programs that are tailored and flexible and involve cooperation between the psychiatric system and social services” (Nordentoft, 1990, p. 439, translated from danish). Such programs have yet to materialize at scale (Johansen and Thylstrup, 2019). While there are presumably also several causes for this lack of holistically oriented services it has been suggested that progress has been hampered by lack of engaging with the breadth of processes involved in a decentered treatment system (Rosenberg and Hickie, 2013; Priebe, 2016). Figure 1 places the important developments in relation to each other.

### A pragmatic approach: Pinpointing a central challenge

So far we have sketched the emergence of the field of dual diagnosis. Even though the preceding pages constitute only one of several possible ways to tell the story, researchers within the field widely support that the emergence of the field of dual diagnosis was connected to or shaped by changes in the diagnostic system, the increased availability of substances, the deinstitutionalisation process and the lack of a timely developed alternative system, and a predominantly biomedical approach to dual diagnosis problems.

Seeing the field of dual diagnosis as fundamentally constituted by this mosaic of processes alerts us to the contextual nature of dual diagnosis problems. As suggested by two veterans in the field of dual diagnosis - a psychiatrist and a psychologist - the concept of dual diagnosis is a “misnomer,” which has both good and bad effects:

“On the positive side, when the complexities were reduced to a simple medical term, attention was drawn to problems related to substance use, which created a mandate for recognition and treatment. However, […] the medical designation also focused attention on the biological and pharmacologic aspects of treatment, implying that substance use problems inhere in the patient and muting the role of public policy in creating such problems” (Drake and Wallach, 2000, pp. 1126–1127).

In other words, dual diagnosis problems tend to be understood in a lopsided way where some aspects are accentuated, while others are set aside. And whereas the above quote relates to the dominance of a biomedical perspective, research focusing on psychosocial aspects also tends to downplay biological aspects (e.g., Drake et al., 2002), thus leaving us without a framework addressing factors across disciplinary boundaries. Returning to our pragmatic approach, which suggested that we should start by examining and defining the difficulty that we wish to address, we can now describe one central challenge of the field of dual diagnosis, i.e., the *lack of an integrative framework for organizing the different kinds of factors impinging on dual diagnosis problems*. Unfortunately, according to our pragmatic approach, no linear, undisputable way of moving from difficulty to possible solution is available. Rather,

“[…] it involves a leap, a jump, the propriety of which cannot be absolutely warranted in advance, no matter what precautions be taken. […] The suggested conclusion so far as it is not accepted but only tentatively entertained constitutes an idea. Synonyms for this are supposition, conjecture, guess, hypothesis, and […] theory” (Dewey, 1910, p. 75).

While diagnoses are usually seen as helpful for organizing treatment, within the dual diagnosis field the possibility exists that the concept of dual diagnosis is part of the problem. In the following, we therefore adopt the Enactive Psychiatry (EP) approach to psychiatric disorders, which (1) is bio-psycho-socially integrative in nature, which (2) applies a perspective in which different factors are seen as dynamically related, and which (3) does not depend on diagnoses *per se*.

We introduce the EP theory of mental disorders through examination of data from a qualitative, longitudinal study of cannabis use in psychosis disorder in order to examine what picture of dual diagnosis this type of approach may help develop. We emphasize again that we do not intend to “prove” the theoretical perspective [as the approach is formulated at the hard core level, it is not directly falsifiable (Wołoszyn and Hohol, 2017)]. Rather, our aim is to examine if this kind of approach may constitute a step toward developing an improved “grip,” in our pragmatic sense (Pickering, 2017, p. 137), on the dual diagnosis phenomena. Furthermore, the presentation of the theory and the empirical study are structured to foreground how theory and empirical data “communicate” and also include references to other research findings in the field to help guide our reading of the empirical material.

## Empirical study: Cannabis and psychosis from the enactive psychiatry approach

**FIGURE 2:**
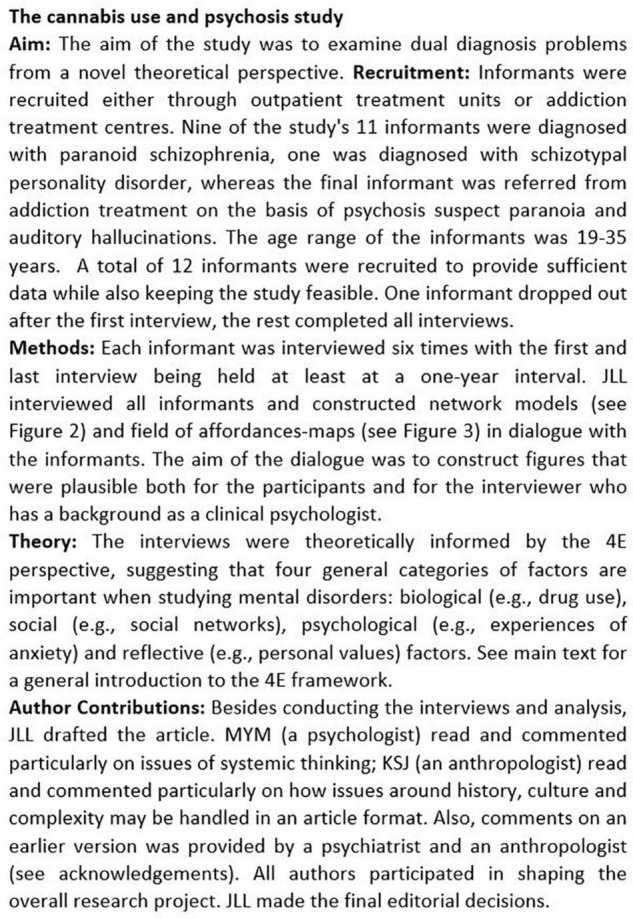
The cannabis use and psychosis study.

**FIGURE 3:**
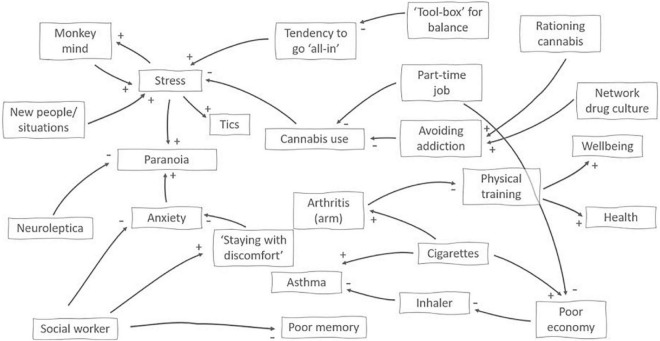
A network visualization of factors impinging on and contextuaIising cannabis use in a 29-year-old woman diagnosed with paranoid schizophrenia. + or– at the end of an arrow indicates the effects of a change in the originating factor, i.e., new people/situation increases stress, whereas meeting with her social worker helps reduce anxiety. The map was developed in dialog with the participant and used the four categories of factors from the 4E approach (Figure 1A above) as a guide.

The empirical material in this article is drawn from a longitudinal, qualitative study examining cannabis use in context of psychosis (see Figure 2 for an overview of study aims and methods). Figure 3 is one the 11 models constructed during the study and it provides an overview of factors involved in a dual diagnosis case. The figure concerns a 29-year-old woman (here called “Louise”) with an extensive history of drug use, now limited to daily cannabis use and monthly use of other drugs such as amphetamines and hallucinogenics. Louise is also diagnosed with paranoid schizophrenia and - like the other ten informants - participated in six interviews held in the course of a year, the aim of which was to collect rich information on:

• Their background
• Emergence of mental health problems
• History of drug use
• Treatment history
• Present life situation (including drug use and mental health concerns)

Through the longitudinal design (Saldaña, 2003), the picture of the participant’s present life situation was then expanded into a developmental history: How do circumstances and concerns change with time? How do plans unfold, what work for them in managing their problems and what does not work? What is expected of the future now versus in a later interview? In collecting and exploring our data, we drew on Enactive Psychiatry (EP) (de Haan, 2020b). EP has roots in the so-called 4E approach to cognition which was initially developed as a reaction to the increasingly disembodied and computational accounts of cognition within the broad field of cognitive science (Varela et al., 1991) - a state not unlike the focus on the brain within brain-disease accounts of mental disorders and addiction (de Haan, 2020a, p. 21, note 3). What the four E’s suggest is that cognition is Embodied, Embedded, Enactive and Extended, which means that we must study not only the brain but also the body; how the body is embedded in the environment, how the person experiences and acts, and how the person uses resources outside the body to accomplish tasks. Furthermore, the approach suggests that a historical perspective is needed: We need to examine the person’s history of engagement with the environment because all organisms have a history of coupling with the environment that lays the ground for their present capacities. On the 4E account, mental disorders are not only biological, only psychological or only social entities. Rather, mental disorders, in the words of the ecologically oriented German psychiatrist Thomas Fuchs, are “the product of a cascade of subjective, neuronal, social and environmental influences continuously interacting with each other.” (2009, p. 230).

Enactive psychiatry is a particular instantiation of the overall 4E paradigm. EP suggests there are four main categories of factors involved in mental disorders: biological, experiential, socio-cultural and existential factors. EP is comparable to the biopsychosocial model (BPSM) however it is better at achieving integration of the factors because - unlike the BPSM (Engel, 1980) - the four categories are understood not as parallel domains “across which material and information flow” (Engel, 1980, p. 537) but as *aspects* of one and the same complex, dynamic process. Biological factors and experiential factors are thus not understood as different kinds of processes but are understood as implying each other: Matter as configured in living systems is considered minded (de Haan, 2020a). Thus, EP cuts across dichotomies prevalent in mainstream thinking on mental disorders (Parnas et al., 2013) which speaks to our search - in context of the dual diagnosis field - for an integrative framework. EP also uses the concept of *sense-making*. Sense-making is the basic process through which living systems keeps themselves alive: they avoid what kills them and pursues what keeps them alive. EP distinguishes between *basic sense-making* - which characterizes all living beings - and *existential sense-making* which characterizes human beings. Existential sense-making refers to the human capacity for stance-taking which opens a new realm of sense-making: For humans everything from the clothes one wear, the food one eats, to how the body looks and so forth becomes imbued with meaning: “With stance-taking a different kind of values emerges, what we could call “existential values,” like respect, honor, dignity, friendship, and love” (de Haan, 2020a, p. 9).

From the EP perspective mental disorders can be seen as disorders of sense-making. When sense-making lacks adequate flexibility and adaptability and the same inappropriate patterns emerge in experience, thinking and/or behavior over and over again it moves into the territory of a disorder. Because sense-making is a process, disorders does not reside in the brain nor in the body, although it does involve both brain, body and the world in which the person lives. Thus:

[…] if we want to understand psychiatric disorders, we should look at persons in interaction with their specific worlds […] [and] look at this person-world system as it has developed and is developing over time to understand its dynamics. (de Haan, 2020a, p. 11, emphasis removed)

In the present study we used the four domains EP suggests as a framework for ordering the empirical material while the analysis was concerned with understanding dynamics and change. The EP framework was thus essentially in line with our qualitative, longitudinal design for which we adopted the following goal:

[…] to capture through long-term immersion the depth and breadth of the participants’ life experiences, and to capture participant change (if any) through long-term comparative observations of their perceptions and actions. (Saldaña, 2003, p. 16)

Furthermore EP also suggests to employ *personalized network models* as a heuristic tool for describing the person-world system and the factors influencing its trajectory. This draws on an interest for understanding mental disorders as self-perpetuating interactions between elements (Roefs et al., 2022). EP specifically suggests constructing personalized network models based on the four general domains in combination with qualitative data (de Haan, 2020b), thus allowing a high degree of sensitivity to the individual case while also placing the model in an integrative theoretical framework. A second mapping from the EP framework is also employed in this study - the so-called Field of Relevant Affordances - which is defined later in context of the empirical material. Both kinds of mapping were done in collaboration with the informants. With the theoretical framework introduced we turn to presenting the empirical material. Additional concepts introduced in the following empirically driven sections are also all connected to the EP framework.

### Curbing consumption: Embedded and extended aspects

When Louise was first interviewed, it became clear that positive changes had recently occurred. Earlier, she had used both amphetamine, cocaine, ketamine and hallucinogenics; she had been severely paranoid and without any concrete plans for her future. At the time of the first interview, however, other drug use was limited; and although cannabis was used daily, she consumed smaller quantities than earlier. Her symptoms, consisting of paranoia and the hearing of whispering voices, was generally under control and favorably influenced by antipsychotic medication. As illustrated in Figure 3, cannabis served a stress-reducing function: It helped her manage invasive thoughts and helped her relax her body. Thus, the use did not directly interfere negatively with other activities. From the EP perspective, we should not expect to find the reason for this ability to reduce and keep her cannabis use stable only in personal attributes such as willpower or grit (although personal strengths may certainly play their part), but also in her social and environmental embeddedness. This is supported by the empirial material where two aspects, in particular, stand out.

Firstly, public job services placed the informant in a time-limited part time job to test her abilities. The informant participated in choosing the job and hoped to be able to either finish an education or to take on a part time job permanently. Attending her job helped her curb her cannabis use because she did not use cannabis before going to work as she acknowledged that being under the influence diminished her performance. Secondly, the informant had the intention of not (again) having her life dominated by drug use. During the period when she was interviewed, she did not see herself as addicted but rather as a rational user. Realizing her intention of staying low on consumption was supported by two factors. One factor was the peers in her social network, who - like herself - were also users of cannabis and occasionally other drugs, but they were also *controlled* users who supported each other in keeping their consumption low. This occurs through not pressuring each other, through example and through a shared interest in educating themselves about how different drugs work and how to use the drugs while minimizing their deleterious effects. The other factor was a cannabis rationing system, which the patient had developed. She bought cannabis once monthly and immediately upon returning home divided it into daily rations, which were wrapped individually in tinfoil and placed out of sight in a bowl. This, she reported, had made it easier for her not to consume excessively because once the daily dosage was used, it required a conscious decision to start using tomorrow’s dose.

This illustrates that strategies for managing drug use according to one’s intention is a complex skill that weaves together formal duties and plans for the future, social relations and manipulating the local environment in order to make unwanted courses of action less salient. Psychiatric treatment also plays an important role. In particular, the informant mentions her long-term participation in a professionally led support group, which she still attends, learning, among other things, emotion regulation skills. However, first and foremost, it shows how decision-making on drug use in the context of psychosis includes an ecological aspect: The volatility of a drug is not determined by the drug’s influence on brain function only; rather, it is situated in a wider network of activities and concerns that may potentially temper its use.

### Gradual shifts in trajectory: Circular network effects

Although dual diagnosis problems are associated with a poorer prognosis (Schmidt et al., 2011) and professionals tend to meet dual diagnosis patients with some resignation (Pinderup, 2018), reasons for optimism appear to exist. In a prospective quantitative study, Drake et al. (2016) concluded that in the longer term - here 7 years – 60% of the patients with a combination of schizophrenia and substance abuse reached the researchers’ definition of clinical recovery. Whereas this may possibly be contributed to treatment effects, the authors point out that the same kind of results have been found among people who received little if any formal treatment, suggesting that there may well be many different paths to recovery and that the role of treatment may be to help laying out these paths (Drake et al., 2016, p. 206).

**FIGURE 4:**
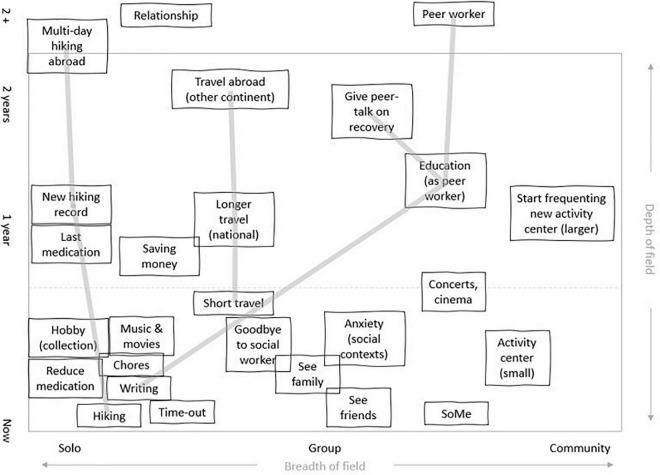
This *field of relevant offordances* from a 31-year-old male diagnosed with paranoid schizophrenia shows the affordances present for one informant when considering both the present and possible future occurrences. The gray lines show examples of congruence between present activities and future plans, e.g., keeping diary/blog about recovery education as peer worker, talks and job activities.

Figure 4 shows how the present and future possibilities at the time of the study were perceived by a participant (here called “John”) diagnosed with paranoid schizophrenia, who had previously abused alcohol to the point of lethal intoxication and smoked large amounts of cannabis. John mostly used hash - but after first staying away from smoking (and alcohol) for several years, he started using cannabis (CBD) oil, which he perceived as helping him handle anxiety. He purchased the product (which is illegal) online and usually took a steady dose, although he did vary the size of the dose when experiencing distress, e.g., due to coming tasks.

The figure draws on the concept of a “field of relevant affordances” (de Haan et al., 2013; Rietveld and Kiverstein, 2014), which denotes the actionable possibilities experienced by a person at a certain point in life. The horizontal axis, as implemented here, moves from affordances that can be exercised alone (such as smoking cannabis and playing computer games), over group-based activities (e.g., educational or sports activities) to situations that involve other people on a larger scale (e.g., going to a concert). The vertical axis indicates time: starting with affordances presently, then in a year, 2 years and finally sometime in the future beyond 2 years. The diagrams were created dialogically during interviews; whenever present activities, near-and long-term plans were mentioned, the interviewer asked the participant to detail these activities and to help place them in the diagram.

It is important to note that the term “affordances” (Gibson, 2015, chapter 8) indicates possibilities that exist at the intersection between the individual and the environment. As members of a given society at a given time point, we all have a general understanding of what it is possible to do - e.g., go to the library, the pool, engage in education, travel, etc. This may be understood as the *landscape of affordances* in a general sense. *The field of relevant affordances*, however, is the composite of affordances that a specific individual perceives given his or her particular history, skills, concerns and environment (de Haan et al., 2013, p. 7). In this sense, a field of relevant affordances is dynamic: “[…] the scope of available affordances at a particular place that are taken into account by the person changes; the time span of the horizon of affordances changes; and there will be shifts in our concerns and, related to that, in the relative salience or relevance of the available affordances” (de Haan et al., 2013, p. 8).

John, as depicted in Figure 4, has access to a wide variety of activities in the present. These include, from left to right, activities at home (e.g., listening to music, writing, doing chores), activities involving individual others as well as groups (e.g., visiting friends, hiking, attending a social psychiatric center) and activities involving large groups of people (going to the cinema and to concerts). Moving up the vertical axis, the informant also has expectations for the future, i.e., things he would like to achieve within the coming years. These activities involve education, longer hiking trips, traveling and reducing antipsychotic medication. Affordances may also be found in the shape of challenges or barriers (e.g., attending a larger, more open rehabilitation center, saying goodbye to his long-term social worker).

The field of relevant affordances diagram is a way to visualize how the here-and-now is contextualized by a future horizon. However, as the field is dynamic, we need to understand the processes shaping the field which means looking at how the person-world system evolved over time. In other words, following the EP perspective suggests we describe the processes that shifted *in casu* this informant from being hospitalized with psychosis, performing self-harm, abusing alcohol and cannabis and considering suicide to having most symptoms under control, using a non-intoxicating cannabis product, and having a rich life, including goals for the future, a strengthened sense of agency and strong coherence between present activities (e.g., weekly day hikes), mid-term goals (multiday hikes on national routes) and long-term goals (doing a long, multiday hike abroad).

In the informant’s description, several defining moments arise during his process of recovery, which spans a ten-year-period. The first moment was moving from supported housing for young people with psychiatric problems to a housing offer for people with epilepsy (which the informant has had since early childhood), which he describes as follows: “It was much calmer and there were no drugs. And the staff knew nothing about schizophrenia, but they started to read up on it.” Another important development was training to take the train without getting off after one stop due to anxiety. Here, the informant described a very gradual process where the relationship to the mentor training with him was accentuated as well as the intense effort it took to expand his zone of comfort. Gaining the possibility of using public transport then opened new possibilities for becoming comfortable with other activities: visiting an old friend, going to the cinema (still with support) and so on. Meanwhile, medication, as the informant described it, “made the floor more solid,” referring to how he experienced early signs of psychosis as a bodily sense of losing ground support, thus enabling him to go further in his explorations. A third important development was learning to take care of himself - to avoid staying too long at a friend’s party or at a family dinner if he felt the early signs that he was losing his footing. A central part of this was being more open, not attempting to endure discomfort without showing signs of it, something which created its own positive feedback loop: It now became possible for others, e.g., family members, to help him notice stress because it had become safer to talk about it.

From the EP perspective, each of these changes may be understood as developments that reconfigured the field of relevant affordances. The first by placing the informant in an emotionally calmer environment without drug use; the second by expanding the choice of possible activities; and the third by making interpersonal space easier to navigate comfortably. Although these developments were spread out over a number of years, they nevertheless left traces - both embodied traces in the form of skills but also as environmental resources to be accessed when needed, e.g., a friend to go on a hike with after a hard week, an empathic call from a close family member and the joy of looking forward to a concert with a favorite band.

These factors are important because they play a part in a larger quilt put together by many different and partly overlapping pieces, where a beneficial element in one domain connects to another in a different domain. From the EP perspective, change is a product of this network of factors - not of central causes. This presupposes a systemic view of effects, where changes in one area (e.g., being more open about feelings, stresses, having stronger “ground support”) reverberate through the environment and back (e.g., allowing new positions in interpersonal space, creating the possibility for more pro-active support from close relatives). Over time, feedback loops like these scaffold new elements in the field of relevant affordances: Medicine initially calms psychosis, making skills training possible, allowing new options, enabling longer term goals; and these goals, in turn, make it meaningful to overcome challenges in the here-and-now, including reducing the initially beneficial medication in order to avoid side effects such as weight gain.

In the literature these kinds of processes are described in various ways. The psychiatrist Thomas Fuchs for example writes about *circular causality*, where the “circular interactions of self, body, brain and environment may be approached at various levels or turning-points, since any mode of treatment will be transformed by the brain and thus contribute to a holistic effect” (Fuchs, 2009, p. 231). The EP perspective, however, instead refers to a *horizontal network structure* serving to avoid reifying a vertical hierarchy of levels (de Haan, 2020a, pp. 223–224). Both nuances combine their view with a distinction between local-to-global effects (e.g., medication leading to experiential and behavioral changes) and global-to-local effects (e.g., participating in psychotherapy leading to neural changes).

When the series of interviews with this informant concluded, he said that he intended to continue using CBD oil, he had reached (and set) new goals in his hiking activities, was saving money for a trip abroad, learning more about his educational possibilities and was expecting to soon discontinue his antipsychotic medication in collaboration with his psychiatrist.

### Qualitative shifts in perspective: Transition to abstinence

To describe our final theme, we will draw on material from several cases. Whereas the above theme saw changes in the field of relevant affordances as gradual reconfigurations, more sudden shifts also occur. Two informants were hospitalized for weeks; one moved to a supported housing, one was involved in a traffic accident and two stopped taking antipsychotic medication on their own. All informants at some point talked about discontinuing or cutting down on their cannabis use either due to its effects, the expenditure, the process of acquisition or because close relatives were critical of their use. Two informants decisively stopped using cannabis; one apparently for good, the other for extended periods of time. Several informants had shorter periods in which they did not use cannabis either out of choice or because it was not available.

The informant who stopped using cannabis throughout the remainder of the interviews, a 29-year-old woman diagnosed with paranoid schizophrenia, explained the change as a consequence of her becoming tired of living with addiction as the centerpiece of daily life. Frequently - such as in the 12-step tradition of addiction treatment (Bateson, 1971) - hitting some kind of bottom is understood as a precursor to change. However, again the EP perspective suggests that we take a broader perspective. Examining the factors surrounding this informant’s decision to stop using cannabis also reveals a number of other factors preparing the way for such a change. Firstly, as mentioned, the informant reported experiencing a loss of meaning when there was no future horizon to look forward to, only one day of smoking cannabis leading to the next while life passed. Secondly, she was inspired by an old friend who had finished an education while also having a psychiatric diagnosis. Thirdly, although the informant was generally critical of her pharmacological treatment, she nevertheless had good relationships with the mental health professionals - as well as with her social worker - and she was engaged in understanding her diagnosis (which she also harbored doubts about). Fourthly, in parallel to her psychiatric treatment, she attended addiction treatment, which involved participating in social and physical activities and learning more about her educational possibilities. How can we understand the sudden shift to not using cannabis against this backdrop of shifting factors and influences?

The EP perspective draws on several theoretical and philosophical strands, one of them is dynamical systems theory (Newen et al., 2018). Dynamical systems theory is an often mathematically oriented approach to understanding how complex systems self-organize, i.e., how complex patterns emerge out of more basic processes without there being a central, organizing hub. However, the approach has also been employed in human developmental studies. Work on infant development, e.g., has described how the novelty of walking is preceded by combining simpler movement patterns in new ways leading to the emergence of a - for the individual - revolutionary new way of moving (Thelen and Smith, 1996). Similarly, the shift to abstinence may be understood as the repurposing of resources in a - for the individual - novel way. Dynamical systems theory posits that complex systems furthermore tend to organize into particular modes of functioning (also called *attractor states*) (Thelen, 2005, p. 264). What is particularly relevant to note is that a change in mode of functioning presupposes that certain, more basic, elements are present.

**FIGURE 5:**
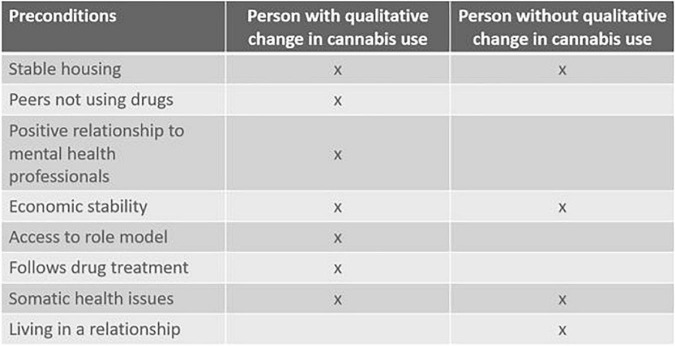
The presence of factors possibly supporting transition to abstinence compared across two cases. The left column describes a 29-year-old female, the right column characterizes a 19-year-old male. Both were diagnosed with paranoid schizophrenia.

A comparative illustration of what an effective difference in preconditions may look like can be made by drawing on another case in which a young man (19, paranoid schizophrenia) also expressed a wish to reduce his daily cannabis use but failed to implement any decisive changes during his participation in the study. Again, an individualist interpretation might point to his lack of character strength, his degree of disorder or the level of his addiction. But the EP perspective again suggests we examine what preconditions exists for this change to be feasible. Here, clear differences exist between the positive and the negative example of a qualitative shift. Figure 5 illustrates differences in preconditions between cases.

Whereas Figure 5 compares two cases chosen for their illustrative potential, it also suggests as a hypothesis a more general pattern, namely that a certain “thickness” of factors pointing into a new field of relevant affordances may be needed to move decisively toward recovery. A similar point was made in a study by Alverson et al. (2000). This study discerned four Positive Quality of Life Factors (having access to an enjoyable activity; decent and stable housing; a caring relationship with an accepting, sober person; and a positive relationship with a mental health professional) as precursors to abstinence in a dual diagnosis population when three or four of these were present. Whether or not these are the exact, universal factors to look for, findings such as these underline the need for coordination and perhaps even timing of different types of interventions and underpin how they should fit the patients’ wider circumstances of life.

## Discussion

The empirical material we have presented is largely of an optimistic nature. It shows how life may change for the better among patients who at one point were severely impaired due to their dual diagnosis. Drug use can be ceased or at least brought within certain limits, social life can be enriched and future prospects may take shape and guide present choices and energize efforts to overcome barriers. The empirical material also contains illustrations of a lack of change, setbacks and periods with severe suffering; cases in which access to social milieus of predominantly serious drug users feed back and disconnect the individual further from non-drug-using relations; or in which the side effects from medication - drowsiness, weight gain, muscle tension - reduce the likelihood of engaging in new experiences and resources; or where the online universe of preachers on YouTube leads down a cannabis-furnished rabbit hole full of conspiracy theories; or where stigmatization alienates patients from close relatives. We have focused on examples of recovery processes that lead to richer lives, but we note, too, that the less fortunate trajectories also present in the material often are similar to how the positive cases fared at an earlier point. Thus, there is hope.

Our theoretically informed examples illustrate some possible paths to recovery - but these paths are not centrally organized and probably cannot be so either. Rather, these paths are fashioned by the patients themselves based on the resources accessible to them. Drawing on the EP perspective, they may be coined paths “laid down in walking” (Varela et al., 1991) - a way of describing developmental trajectories with a foregrounding of process, of doing.

We have examined what patterns and processes a complexity-oriented and ecologically oriented theoretical approach help us acknowledge in qualitative, longitudinal data from the field of dual diagnosis. Particularly, we have seen how changes in the lives of complex dual diagnosis patients may be understood as ecologically embedded processes in which recursiveness between the individual and his or her environment plays a crucial role. Psychiatric treatment - medication, sometimes therapy or psychosocial interventions - plays an important part, too. Over time, these diverse influences shape what the person experiences as relevant out of the whole landscape of affordances that the milieu offers. The EP framework also suggests two paradigmatically different ways of performing interventions: either as a local-to-global process or as a global-to-local process. Seeing the gap we have described between a biomedically oriented psychiatry and a psychosocially oriented addiction treatment from this perspective allows us to recognize both simultaneously.

This insight is echoed in the pragmatic approach to knowledge. The philosopher of science Thomas Kuhn famously described scientists on opposing sides of a paradigm shift as practicing “their trades in different worlds” (Kuhn, 1996, p. 150). But, from our pragmatic point of view, differing explanations may co-exist without conflict provided we understand knowledge as tied together with performance, with doing things (Pickering, 2017, pp. 135–136). Focusing on neural reward structures is simply one way of attempting to get a “grip” (Pickering, 1995, p. 188) on addiction problems. The same goes for psychosocial interventions and the theories that come with it. In an ontological sense, both substance use and mental disorders are phenomena of such richness that they can be engaged with (potentially effectively) in various ways - through biological intervention, through psychotherapies, through environmental interventions, through social networks as Marijuana Anonymous, Dual Diagnosis Anonymous (Milani et al., 2020) and through policy changes, etc.

Even so, a framework for integrating the different types of “grip” on dual diagnosis problems is needed to facilitate cooperation between disciplines and service providers. Our experiment with examining cannabis use in the context of psychosis disorders through the EP lens alerted us to the possibility of synergistic effects - either as part of a continuous recovery process or as a precondition for shifts in mode of functioning. Here, the EP perspective provided us with a way of developing a dialogue between the different professional grips on dual diagnosis problems as well as with the patients’ own grasp of their situation. Thus, the EP perspective may provide a way to conceptually and methodologically support the emerging recovery-orientation within the dual diagnosis field (van der Stel, 2015; Brekke et al., 2017).

We can exemplify what the EP approach offers by comparing it to another line of thinking in psychiatric research: Whereas it has been repeatedly shown that many patients with schizophrenia exhibit self-disturbances (Henriksen et al., 2021), which is sometimes understood as the “essence” of the disorder (Kyselo, 2016), this phenomenological approach - while valuable and sophisticated - does not attempt to address the diverse factors involved in psychiatric disorders and even less so in dual diagnosis cases. It provides insight into one dimension but does not help us understand the whole situation or weigh different possibilities for intervention against each other (de Haan, 2020b, pp. 17–18). In contrast, the EP approach shifts to a multidimensional perspective and seeks to comprehend how different dimensions (which may importantly include self-disturbances) interweave. This speaks to our concern about creating synergy between services and treatment, life circumstances and the experience of the patient.

To sum up the reviewed theory and empirical material: Adopting the Enactive Psychiatry perspective we have used *personal network models* (PNM) and the *field of relevant affordances* (FoRA) to structure our data. PNM models how interacting factors maintains mental disorders, and it draws on an EP version of the biopsychosocial model that is well suited to incorporate qualitative data (de Haan, 2020b). The FoRA is informed by phenomenology and maps the self-world relation in an ecologically atuned manner (de Haan et al., 2013). Both the EP approach and the particular methods of PNM and FoRA are relational in the sense that neither organism nor environment are considered separately but instead understood as intertwined at the root. Although representing a challenging way of thinking because it goes against an ingrained tendency to reify processes into localizable essences, the EP approach makes it possible to arrive at a contextual model illustrating how different disciplines can meet. Following the EP approach, knowledge about neural, psychological and socio-cultural processes are all relevant. However, what this knowledge *means* cannot be understood separately from the whole. We suggest that approaching the dual diagnosis field from the EP perspective adds an integrative understanding of the person-in-context that has so far been missing from the field (Drake and Green, 2013).

We have made two specific suggestions for studying dual diagnosis problems in a different light. Firstly, we suggested attending to feedback loops that, over time, shift the patient’s field of relevant affordances in a beneficial way. These feedback loops may be understood only through a cross-disciplinary lens because they involve the reverberation of changes in one context (e.g., receiving medication in an adequate dose) through other contexts (e.g., being able to engage more fully in a psychosocial training). Identifying and describing particular and/or typical feedback loops at different levels of analysis may provide a fuller picture of how recovery from dual diagnosis unfolds and perhaps how it may be supported more robustly. Secondly, we suggested that a certain thickness of factors pointing into a changed landscape of relevant affordances may be necessary to shift into a more beneficial trajectory of recovery. This hypothesis, as referenced, is not new, but the EP framework provides some conceptual tools allowing us to describe these changes in more detail - for example in the context of a quantitative or mixed-method type of research.

### The new theories and psychiatry

Whereas the EP framework may inform a progressive problemshift (Lakatos, 1980) within the dual diagnosis field, this is not necessarily the case for psychiatry in a wider sense. As mentioned in the introduction, recent years have seen different researchers engage in the same kind of challenges that we have described within the field of dual diagnosis but focusing on the whole field of psychiatry. In closing, we therefore want to consider the EP approach and the similarly 4E-related *neuroecosocial mental health* (Rose et al., 2021), *ecosocial psychiatry* (Kirmayer, 2019) and the *relational analysis of phenomena* (Nielsen and Ward, 2020) under one as a group of *new hard-core theories* of mental disorder in the Lakatosian sense, which may be frontrunners for a future broad problemshift within psychiatry. The new theories include the ecological aspects of mental disorders, gives particular attention to integrating the perspectives of different disciplines and methods, and draws on systems thinking. Even so, as described historically and empirically, dual diagnosis involves many factors in complex interaction and involves integrative cooperation between disciplines, which may make the new theories particularly useful for this clinical context. For people with less complex problems, achieving change may be supported without engaging so extensively with complexity, e.g., by using monodisciplinary (such as psychopharmacological or psychotherapeutic) interventions to remove barriers for engaging more fully with otherwise already beneficial factors in life.

However, a wider potential may exist. One proponent of the new theories, the transcultural psychiatrist Laurence Kirmayer^1^, provides us with a speculative image of what the future may hold, by imagining that the mental health professionals of the future may work as follows: “[…] we need a nosology based not just on neural circuits but on personal and social predicaments. The choice of level of explanation and intervention then will be based […] on pragmatic decisions about where clinical leverage can be found” (Kirmayer and Crafa, 2014, p. 10). In the same vein, Nielsen and Ward (2020) suggest looking for “engines of distress,” which are self-reinforcing feedback loops involving organism and environment, e.g., as in anxiety leading to avoidance, which deepens the rut while, over time, diminishing the salience of alternative actions through lost skills, relationships and so forth (within our particular dual diagnosis focus on long-term trajectories, we suggest complementing this with a positive concept such as “engines of rehabilitation”). Similarly, Rose (2016) suggests initiating treatment planning as well as research from accounts of adversity at the personal level before proceeding to examine how this adversity “may get under the skin,” something which also suggests a collaborative process involving professionals from various disciplines as well as the patients themselves.

It has been suggested that present diagnostic systems are less useful for guiding clinical practice than has been routinely assumed. This rests on a distinction between the initial categorical diagnosis of a patient and the process of describing the actual, unique patient and then preparing a treatment plan:

“[…] up to now, the first step (diagnosis) has received a lot of attention, with the production of several generations of tools providing systematic guidance to the clinician, whereas the second step (further characterization of the individual case) has been largely ignored […]” (Maj, 2018).

The new theories with their foregrounding of processes, feedback loops and attentiveness to the experience of the individual may be well suited to contribute to fill this gap. However, as has been attempted in this paper, from a pragmatic perspective, any wider utility must be examined by addressing concrete difficulties encountered in the mental health field if the new theories are to achieve an impact on clinical practice.

### Summary

Starting from the historically informed challenge of establishing an integrative framework for the cross-disciplinary and cross-sectoral field of dual diagnosis, we used empirical data to examine if dual diagnosis problems may be favorably understood by adopting the Enactive Psychiatry perspective which sees psychiatric disorders as mosaics of situated processes traversing the disciplinary boundaries of biology, psychology and sociology. The empirical analysis suggests that these mosaics may meaningfully be visualized as network structures and fields of affordances informing our understanding of what promotes or impedes rehabilitation. Whereas the specifics of the empirical analysis are unique and focus narrowly on cannabis use and psychosis, the general thrust of the presented approach suggests that the EP approach - or an amalgam of the “new theories” - may be developed into a combined theory-and-research framework catering to the integrative needs of the field of dual diagnosis in order to support long-term trajectories of recovery. Regarding the potential wider impact of the EP approach and the related ‘new theories’ it may rest on their ability to supplement existing diagnostic systems by providing innovative tools for characterizing individual cases in order to improve treatment.

## Data availability statement

The datasets presented in this article are not readily available because of data protection requirements. Requests to access the datasets should be directed to JLL, jonathan.led.larsen@regionh.dk.

## Ethics statement

Ethical review and approval was not required for the study on human participants in accordance with the local legislation and institutional requirements. The patients/participants provided their written informed consent to participate in this study. Written informed consent was obtained from the individual(s) for the publication of any potentially identifiable images or data included in this article.

## Author contributions

Besides conducting the interviews and analysis JLL drafted the article. MYM (a psychologist) read and commented particularly on issues of systemic thinking. KSJ (an anthropologist) read and commented particularly on how issues around history, culture, and complexity may be handled in an article format. All authors participated in shaping the overall research project.

## Conflict of interest

The authors declare that the research was conducted in the absence of any commercial or financial relationships that could be construed as a potential conflict of interest.

## Publisher’s note

All claims expressed in this article are solely those of the authors and do not necessarily represent those of their affiliated organizations, or those of the publisher, the editors and the reviewers. Any product that may be evaluated in this article, or claim that may be made by its manufacturer, is not guaranteed or endorsed by the publisher.

## Footnotes

1 The title of the present article is inspired by the cited publication.
